# Supplementary material for: Mapping the evolution of fertility support policies in China: A content and instrumental analysis
Source: PLoS One. 2025 Oct 9;20(10):e0332137. doi: 10.1371/journal.pone.0332137 (PMC12510515; doi:10.1371/journal.pone.0332137)
Supplement: S1 Appendix — (ZIP) [file pone.0332137.s001.zip › S1 Appendix. 226 original policy documents/2-国家计生委、民政部关于加快城市社区人口与计划生育工作改革的意见(FBM-CLI-4-38798).doc]

国家计生委、民政部关于加快城市社区人口与计划生育工作改革的意见

**发布部门：** [国家计划生育委员会(已更名)](https://www.pkulaw.com/law/chl?Aggs.IssueDepartment=60314) [民政部](https://www.pkulaw.com/law/chl?Aggs.IssueDepartment=60208) [机构沿革](javascript:void(0))

**发布日期：**2001.10.31

**实施日期：**2001.10.31

**时效性：** [现行有效](https://www.pkulaw.com/law/chl?Aggs.TimelinessDic=01)

**效力级别：** [部门工作文件](https://www.pkulaw.com/law/chl?Aggs.EffectivenessDic=XE0303)

**法规类别：** [人口计划管理](https://www.pkulaw.com/law/chl?Aggs.Category=09701)

国家计生委、民政部关于
加快城市社区人口与计划生育工作改革的意见
（２００１年１０月３１日）

各省、自治区、直辖市、计划单列市、新疆生产建设兵团计生委、民政厅（局）、计生协：
　　为贯彻落实《[中共中央国务院关于加强人口与计划生育工作稳定低生育水平的决定](https://www.pkulaw.com/chl/f827fcaffffe8d79bdfb.html)》和《中共中央办公厅国务院办公厅转发〈民政部关于在全国推进城市社区建设的意见〉的通知》精神，加快社区人口与计划生育工作改革，建立适应社会主义市场经济和社区建设要求的管理体制，进一步提高城市计划生育工作水平，现提出如下意见：
　　一、充分认识加快城市社区人口与计划生育工作改革的重要性和必要性
　　大力推进城市社区建设是城市管理体制改革的重要内容，是新形势下坚持党的群众路线，做好群众工作，加强基层政权建设和社会主义民主政治建设，加快城市现代化进程的战略性任务，对于促进城市经济社会协调发展，提高人民的生活水平、生活质量和社会文明程度，扩大基层民主，维护社会稳定，推动城市改革与发展，具有重要意义，也为稳定城市低生育水平、综合治理人口问题创造了有利条件。
　　社区建设的基本原则是以人为本、服务居民。稳定低生育水平，提高人口素质和居民的生活质量，是社区建设的重要目标之一。要充分认识做好城市社区人口与计划生育工作的重要性和必要性，正确处理社区建设与计划生育工作、履行政府职能与推进居民自治、依法管理与改善服务、发挥部门优势与合理利用社区资源的关系，促进人口发展与社区建设良性互动。多年来，城市人口与计划生育工作始终走在全国前列，为控制人口增长，提高人口素质做出了重要贡献，对广大农村地区起到带头、示范和辐射作用。随着社会主义市场经济体制建立和城市化进程加快，大量流动人口进入城市，许多“单位人”变为“社会人”，政府和企事业单位的一些社会职能逐渐向社区转移，给城市特别是社区人口与计划生育工作带来新的压力。原有以“条条”和单位为主的管理已不适应日益开放、动态的运行体系，城市居民对计划生育咨讯和技术服务的需求不断增长，低生育水平下的人口问题，如独生子女教育培养、老龄人口的养老、生殖健康和性教育问题日趋突出，人口与计划生育工作的难度加大，要求更高，任务更艰巨。
　　社区建设给城市人口与计划生育工作带来新的机遇和挑战。抓住这个机遇，推动城市人口与计划生育工作融入社区、服务社区，建立与之相适应的管理体制和运行机制，进一步巩固和加强社区基层基础工作，增强服务性、群众性和管理的科学性，是城市人口与计划生育工作综合改革的必然要求，是稳定低生育水平，坚持以人的全面发展为中心，推进人口问题综合治理，加快工作思路和工作方法“两个转变”的当务之急。

　　二、明确城市社区人口与计划生育工作改革的指导思想和目标
　　城市社区人口与计划生育工作的改革创新，要以邓小平理论和江泽民总书记“三个代表”重要思想为指导，依托社区，建立完善与社会主义市场经济体制相适应的人口与计划生育管理体制和相应的保障制度；坚持以人为本，注重人的全面发展，尊重社区居民的计划生育主人地位，最大限度地满足社区居民在计划生育、生殖保健、家庭社会化服务等方面日益增长的需求；落实计划生育基本国策，稳定低生育水平，提高人口素质，促进城市社区人口与经济社会协调发展，促进社区的物质文明和精神文明建设。
　　“十五”期间，城市社区人口与计划生育工作改革的目标是：
　　（一）按照建立适应社会主义市场经济体制要求的“依法管理、村（居）民自治、优质服务、政策推动、综合治理”的人口与计划生育工作管理机制的总体要求，在党的领导和政府指导下，依托社区，建立“属地管理、单位负责、居民自治、社区服务”的城市人口与计划生育管理机制，不断增强社区计划生育管理服务能力和自治能力。
　　（二）制定和完善相应的法律法规和社会经济政策，建立计划生育利益导向和保障机制。坚持依法行政，正确执法，文明执法，形成规范、科学的管理制度、工作程序和质量标准。推行计划生育政务公开、居务公开、民主管理和民主监督。引导社区居民依法履行计划生育义务。
　　（三）依托社区，建立资源共享的计划生育服务体系。全面推进计划生育优质服务，开展避孕节育措施知情选择，确保育龄群众普遍享受较好的生殖保健服务，出生人口素质、社区居民生殖健康水平和生活质量明显提高。
　　（四）计划生育工作与社区精神文明建设紧密结合，深入开展“婚育新风进万家”活动，发展人口文化事业，建设社会主义生育文化。社区居民实行计划生育的自觉性进一步提高，形成少生优育、男女平等、尊老爱幼、家庭和睦的良好风气和新型社区人际关系。
　　（五）健全城市基层计划生育管理和服务网络，发展计划生育协会组织，壮大志愿者队伍。不断改善基层计划生育干部队伍和计划生育协会队伍结构，使之成为具备较高素质和群众工作水平的社区工作者。
　　我国城市之间社区发展水平不同，人口与计划生育工作的差异性大，推进城市社区人口与计划生育工作改革，必须因地制宜，有计划、按步骤地稳步推进。鼓励社区从实际出发，大胆尝试，大胆创新。切实加强分类指导，防止一哄而起，不搞一个模式。

　　三、建立适应城市社区发展的人口与计划生育工作管理机制
　　管理机制是人口与计划生育工作运行的基础，是城市人口与计划生育工作综合改革的重要方面。必须适应改革开放和社区建设的要求，把人口与计划生育工作重心放在社区，落实到社区，服务到社区。在党的领导和政府指导下，建立“属地管理、单位负责、居民自治、社区服务”的人口与计划生育管理机制。
　　“属地管理”是指城市区、街道统一管理驻本辖区各国家机关、社会团体、企事业单位以及工作、生活在本辖区的常住人口、暂住人口的计划生育工作，承担宣传动员、监督检查、组织协调、综合服务的职能。城市基层各级党委、政府对本辖区人口与计划生育工作切实负起领导责任。社区居委会依据国家法律法规和政策，协助政府做好本辖区内所有单位和居民的计划生育工作。
　　“单位负责”是指所有国家机关、社会团体、企事业单位都要依照国家法律法规和政策，认真执行计划生育基本国策，履行各自职责，共同参与对人口问题的综合治理。实行企事业单位人口与计划生育法定代表人责任制，做好本单位的人口与计划生育工作，落实计划生育奖励和优待等有关政策和保障措施，支持、协助和参与社区计划生育管理服务。
　　“居民自治”是指社区居民自治组织和社区居民，依照国家法律法规和现行生育政策规范生育行为，参与旨在建立健康幸福家庭的人口与计划生育方案，组织起来实行计划生育自我管理、自我教育、自我服务和自我监督。将计划生育纳入社区管理服务体系，积极协助政府做好本社区内的人口与计划生育行政管理工作，对政府有关人口与计划生育的行政管理和社会服务进行民主监督。
　　“社区服务”是指依托社区、面向家庭，广泛开展的计划生育综合服务。包括人口与计划生育宣传教育服务，相关法律法规政策咨询和援助服务，避孕节育、优生优育、孕情访视、生殖保健、紧急避孕、性健康和中老年咨询保健等技术服务，独生子女照料等家政服务，为计划生育家庭提供优先优惠的便民利民服务，配合“星光计划”实施，针对老年人群的社会救助和社会化服务，以及组织社区居民开展的多种家庭互助活动。
　　要坚持政府指导与居民自治、改善管理与加强服务、公民履行计划生育义务与居民自治的高度统一。在党的领导和政府指导下，调动方方面面积极性，依托社区开展人口与计划生育工作，寓管理于服务之中，资源共享，综合治理，协调互动，确保城市人口与计划生育工作管理体制的有效运行。

　　四、城市社区人口与计划生育工作改革的基本要求
　　（一）全面贯彻落实计划生育以宣传教育为主、避孕为主和经常性工作为主的方针，进一步加强社区计划生育网络建设、阵地设施建设和各项基础工作，把人口与计划生育工作纳入社区管理和服务体系。城区、街道建立社区人口与计划生育领导小组。社区居民委员会下设计划生育工作委员会。居民委员会成员兼任计划生育委员。有条件的地方可配备专职计划生育委员，并实行“费随事转”。
　　积极推进计划生育政务公开和居务公开，动员社区居民广泛参与人口与计划生育的民主决策、民主管理和民主监督，深入开展社区居民评议计划生育工作的活动，把社区居民的参与程度和满意程度作为评估计划生育工作好坏的重要标准。
　　（二）人口与计划生育工作是社区精神文明建设的重要内容，要将其融入社区文化建设，与“文明街道”、“文明社区”、“文明家庭”的创建活动紧密结合。利用社区资源开展计划生育宣传咨询服务，多形式、多渠道进行经常性的人口与计划生育基础知识教育，普及计划生育、生殖保健、老年保健和青少年性健康等科学知识。深入开展“婚育新风进万家”活动，倡导科学、文明、进步的婚育观念，建立新型社区人际关系。利用社区教育资源，办好社区“人口学校”，改进信息传播方式和渠道，增强人口与计划生育宣传教育的针对性、吸引力和感染力。
　　（三）加强社区对流动人口的计划生育管理。在当地政府领导下，由相关部门、街道和社区组成流动人口管理协调机构，依托流动人口管理服务中心、办证中心、社区警务室、物业管理机构和社区居民委员会，对流动人口实行统一管理。加大对流动人口计划生育管理服务经费的投入。社区居民委员会协助有关部门查验流动人口婚育证明，掌握育龄人员的计划生育情况，加强与流动人口户籍所在地的联系，向流动人口提供法律政策、宣传教育、避孕节育、生殖保健、劳动就业、生产生活、子女入托入学等方面的综合服务，并对有关部门和单位综合管理流动人口计划生育工作进行监督和评议。
　　（四）大力加强计划生育协会组织和社区志愿者队伍建设。计划生育协会组织和社区志愿者队伍在社区计划生育工作中占有重要地位。社区建设为基层计划生育协会提供了广阔的发展空间，赋予了新的历史责任。计划生育协会组织应充分利用社区资源优势、人才优势、技术优势，加强队伍培训，改善队伍结构，改进活动方法，深入开展“我为协会添光彩、我为国策做贡献”活动。办好“会员之家”。按照群众组织的特点开展多种活动，创造健康、活跃的组织形式。协同工会、共青团、妇联、残联、老龄协会等群众团体，动员社区居民参与计划生育，充分发挥其在人口与计划生育工作中的带头、宣传、服务、交流和监督作用。
　　（五）适应人口与计划生育工作属地管理要求，实行育龄夫妇按政策生育，简化审批手续。将具有常住趋势的流动人口纳入现居住地统计，逐步做到由现居住地按政策安排生育。全面开展避孕节育措施知情选择。精简统计报表内容，减少全国范围的、由基层单位逐人、定期采集并逐级汇总的指标。建立与区、街道、社区、相关部门和驻辖区单位相衔接的育龄妇女信息系统。
　　各级计划生育部门要切实转变职能，减少行政干预，把主要精力集中到对社区人口与计划生育工作的政策支持、信息引导、综合协调和提供服务上来，加大对社区的指导力度，研究解决社区存在的实际困难和问题，为社区开展人口与计划生育工作创造良好的政策环境、法制环境。

　　五、建立完善社区人口与计划生育工作的保障机制
　　推进社区人口与计划生育工作改革，关键在于党和政府的坚强领导。各级党委、政府和基层党组织要按照中央要求，把做好人口与计划生育工作摆到可持续发展战略的首要位置，认真研究新情况、新问题，统筹规划并组织实施城市人口与计划生育工作的综合改革，制定有利于加强社区人口与计划生育工作的政策措施，切实做到责任到位、措施到位、投入到位。
　　进一步明确各级政府、各有关部门和企事业单位人口与计划生育工作职责，加强督促，抓好落实。协调有关部门制定和完善有利于计划生育的社会经济政策，为实现人口与计划生育工作属地管理提供政策支持、工作指导、组织和经费保障，指导社区建立由相关部门、企事业单位和社会团体共同参与的联席会议制度。改革完善人口与计划生育工作目标管理责任制，推行计划生育项目管理。建立科学的指标体系和激励机制，促进以考核为主向评估为主过渡。
　　国家计生委、民政部、中国计生协组成指导小组，加强对社区人口与计划生育工作改革的指导和组织协调。各省、自治区、直辖市、计划单列市和省会城市，也要成立相应的领导协调机构，制定社区人口与计划生育工作改革的整体规划和政策措施，并组织实施。各级计划生育部门、民政部门、计划生育协会组织要认真履行各自职责，加强合作，相互支持，共同指导社区做好人口与计划生育工作。
　　高度重视社区计划生育网络和队伍建设。选拔、推荐得力干部抓计划生育工作，落实人员、任务和报酬，从政治上、生活上关心爱护社区计划生育干部，加强对他们的指导和帮助，为他们开展工作创造条件。根据实际情况，从省、市、区计划生育管理和服务机构抽调干部分期分批到社区帮助工作。
　　加大政府对社区人口与计划生育管理服务的公共投入。按照“费随事转”的要求，以项目拨款、政府采购、市场营销、以奖代补等方式，保证社区计划生育工作的必要经费，不断提高社区计划生育管理服务的装备水平。动员驻区单位和民间社会团体为社区计划生育工作提供人力、物力和财力支持。探索和建立人口与计划生育公益基金，鼓励民间捐资、社会募捐和国际捐赠，形成多渠道的筹资体制，确保社区人口与计划生育工作健康稳步地向前发展。
c31344--020307ckj

© 北大法宝：（[www.pkulaw.com](https://www.pkulaw.com/)) 专业提供法律信息、法学知识和法律软件领域各类解决方案。北大法宝为您提供丰富的参考资料，正式引用法规条文时请与标准文本核对。
欢迎查看所有[产品和服务](https://www.pkulaw.com/helps/2.html)。法宝快讯：[如何快速找到您需要的检索结果？](https://www.pkulaw.com/helps/70.html)[法宝](https://www.pkulaw.com/helps/69.html)V6有何新特色？

扫描二维码阅读原文

**原文链接：**<https://www.pkulaw.com/chl/d2518ec5480c571ebdfb.html>
